# Unclassifiable short-rib thoracic dysplasia diagnosed using targeted gene panel sequencing

**DOI:** 10.1038/s41439-024-00302-y

**Published:** 2024-12-03

**Authors:** Erika Nakajima, Yuko Yokohama, Saori Sugiyama, Mio Taketazu, Kenrokuro Mitsube, Takahiro Yamada, Anna Hammarsjö, Giedre Grigelioniene, Gen Nishimura, Yoshio Makita

**Affiliations:** 1 Department of Obstetrics and Gynecology, Asahikawa-Kosei General Hospital, 1-24-111, Ichijo-dori, Asahikawa, Hokkaido Japan; 2 Department of Perinatal Medical Center, Asahikawa Medical University Hospital, 2-1-1-1 Midorigaoka-Higashi, Asahikawa, Hokkaido Japan; 3 Department of Pediatrics, Asahikawa-Kosei General Hospital, 1-24-111, Ichijo-dori, Asahikawa, Hokkaido Japan; 4 Division of Clinical Genetics, Hokkaido University Hospital, Sapporo, Japan; 5 Japan Skeletal Dysplasia Consortium, Tokyo, N14W5, Kita-ku, Sapporo, Hokkaido Japan; 6 Department of Molecular Medicine and Surgery, Karolinska Institutet, Stockholm, Sweden; 7 Department of Clinical Genetics and Genomics, Karolinska University Hospital, Karolinska University Hospital, Gävlegatan 68, 171 76 Solna, Stockholm, Sweden; 8 Department of Radiology, Musashino-Yowakai Hospital, Tokyo, Japan; 9 Japan Skeletal Dysplasia Consortium, 2-1-33, Midorityo, Musashino, Tokyo, Japan; 10 Department of Genetic Counseling, Asahikawa Medical University Hospital, 2-1-1-1 Midorigaoka-Higashi, Asahikawa, Hokkaido Japan

**Keywords:** Disease genetics, Mutation, Genomic analysis

## Abstract

We report a case of a fetus with short-rib thoracic dysplasia (SRTD) with polydactyly that also presented with atypical severe acro-mesomelic ossification defects. Genetic analysis using massively parallel sequencing of a skeletal dysplasia panel revealed compound heterozygous variants in *DYNC2H1*. This clinical report highlights the challenges associated with diagnosing the diverse phenotypes in the SRTD group and emphasizes the importance of genetic surveillance with a targeted gene panel for accurate diagnosis.

Short-rib thoracic dysplasia (SRTD) is a group of autosomal recessive disorders classified as skeletal disorders caused by abnormalities in cilia or ciliary signaling^1^. The characteristic findings include a narrow thoracic cage and short limbs with severe brachydactyly^2–5^. The narrowness of the thoracic cage is associated with death in infancy in affected individuals. In addition, SRTD data may include various visceral anomalies^3^. The diagnosis of SRTD is based on skeletal radiography and clinical findings. In the context of prenatal diagnosis, three-dimensional helical computed tomography^4^ or ultrasonography^6,7^ is also useful for detecting skeletal changes and internal malformations. Clinical and molecular studies of SRTD have revealed phenotypic diversity and overlap due to pathogenic variants in several genes^3,8^. In addition, SRTD patients may present several unusual phenotypes, and the genotype–phenotype correlation may be complicated by triallelic inheritance. Herein, we report a case of a terminated fetus whose skeletal abnormality was diagnosed as unclassifiable SRTD with unexpected molecular findings of biallelic variants in *DYNC2H1*, the most common disease-causing gene of SRTD.

A 36-year-old primipara woman was referred to the hospital at 15 weeks of gestation. Ultrasonography revealed extremely short limbs in her fetus. The femur measured 6.4 mm in length (−5.1 SD), the humerus was 7.4 mm (−6.1 SD), the tibia was 4.9 mm (−6.6 SD), and the radius was 5.0 mm (−6.2 SD) (SD presented is based on the Japanese fetal ultrasound reference^9^). The ulnae and short tubular bones were not identifiable. Other findings included a small thoracic cage, a single ventricle with pulmonary artery atresia, hyperechogenic kidneys, and megacystis. The pregnancy was terminated at 17 weeks of gestation. The terminated baby presented an extremely small thoracic cage, short arms and legs, especially distal segments, postaxial polydactyly, and brachydactyly (Fig. 1, left). Postmortem radiographs revealed generalized skeletal dysplasia with severe immaturity of the skeleton (Fig. 1, right). The thorax was severely narrow with short ribs. The ossification was defective in the vertebral bodies and ilia and absent in the pubic and ischial bones. The overall pattern was similar to that of SRTD types 3 or 1 caused by pathogenic variants in *DYNC2H1, IFT80, WDR34, WDR60*, and *DYNC2LI1*^1,4,5,7,10,11^. In a normal fetus, ossification of the long bones is observed at 15 weeks of gestation, and the ulnae, fibulae, and short tubular bones are visible via X-ray^12^. However, in this case, the ossification of the ulnae and the fibulae was particularly immature, and the ossification of the short tubular bones was also absent. The selective severe ossification defect in the acro-mesomelic segments was atypical; thus, a tentative diagnosis of unclassifiable SRTD was made^8,13^.

**Fig. 1:**
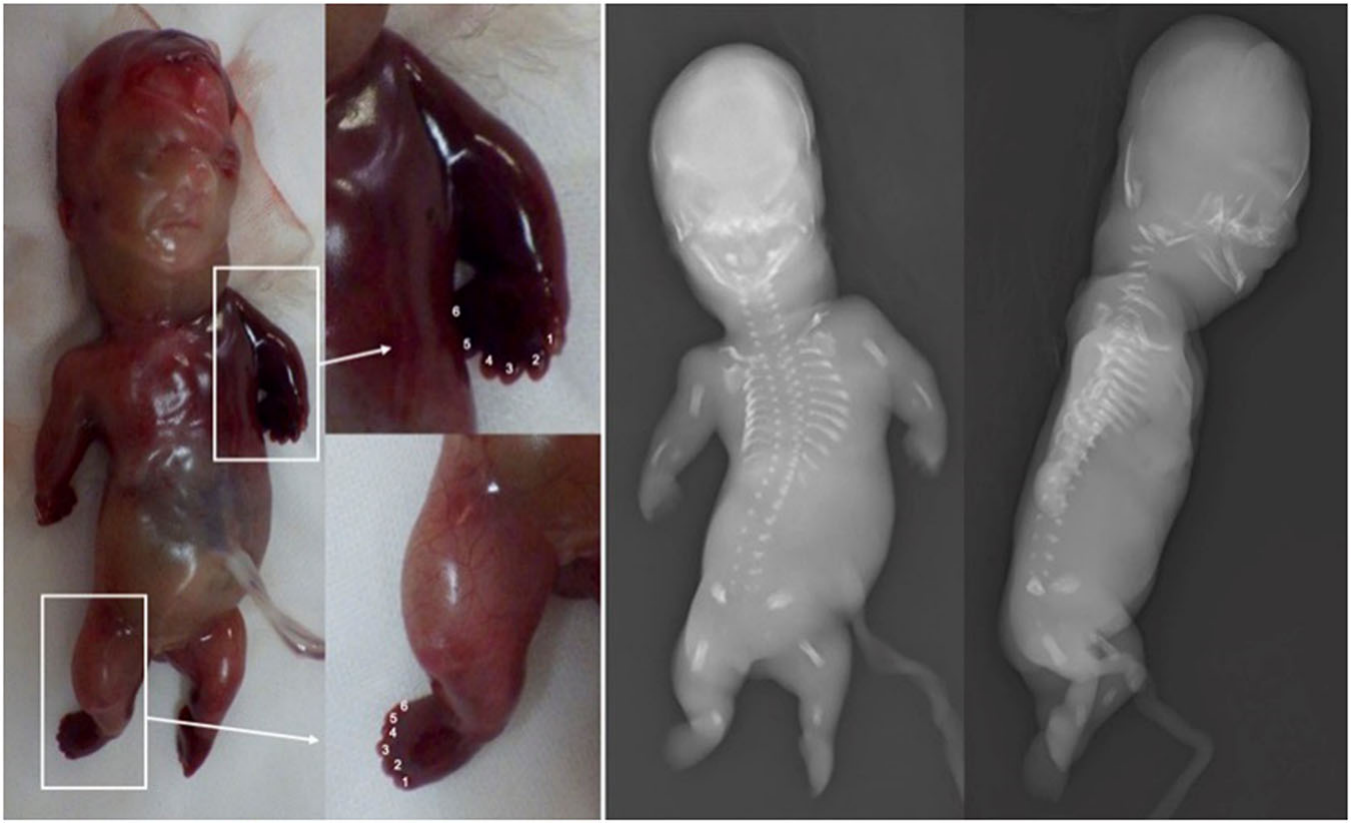
Left: The terminated baby presented an extremely small thoracic cage and short arms and legs, especially distal. Postaxial polydactyly and brachydactyly in the hands and feet are denoted with arrows. Right: Postmortem radiographs of the fetus showing generalized skeletal alterations with striking immaturity of the skeleton, including a severely narrow thorax with short ribs, defective ossification of the vertebral bodies and ilia, and absent ossification of the pubic and ischial bones. The long bones are severely short. The ulnae are particularly short, and the fibulae are not ossified. Ossification of the short tubular bones is absent.

We first performed clinical whole-genome sequencing (automated TruSeq DNA PCR-free library) on DNA obtained from the peripheral blood of the parents. Segregation analysis was performed via exome sequencing of fetal chorionic villi. Both fetal and parental DNA samples were sequenced on an Illumina NovaSeq 6000 (Illumina) with an average coverage of at least 30x at Clinical Genomics Science for Life Laboratory (Stockholm, Sweden) and analyzed in Scout using a targeted gene panel with known skeletal ciliopathy genes^14^. Variants were ranked according to inheritance pattern, variant effect on protein, location in the genomic sequence (encoding exons ±20 bp of intronic sequence), pathogenicity score, and minor allele frequency (MAF) <0.005 according to public and local databases.

We identified four variants in the fetus, including (1) compound heterozygous variants in *DYNC2H1* (NM_001080463.2: c.3404 A > T (p.Glu1135Val) and c.5682_5683del (p.His1896Tyrfs*9), (2) a heterozygous variant in *IFT140* (NM_014714.4: c.3602 G > A (p.Arg1201His) inherited from the mother, and (3) a heterozygous variant in *IFT122* (NM_018262: c.457 A > C (p.Asn153His) inherited from the father. Pathogenicity scores and ClinVar numbers of the variants are presented in Table 1.

**Table 1:** Summary of the detected variants in the skeletal ciliopathy panel.

No.	DNA change	Protein change	Inheritance	ClinVer ID	CADD	Polyphen-2	SIFT	PROVEAN	M-CAP	Frequency (in Japanese population)#	Evaluation of pathogenicity (ACMG/AMP)
1	*DYNC2H1* (NM_001080463.2) c.3404 A > T	p.Glu1135Val	M	SCV005077956	24.8	0.089 (Benign)	0.013 (Deleterious)	−5.45 (Deleterious)	0.096 (Possibly Pathogenic)	—	Likely Pathogenic Previously not reported (PM2, PM3, PP3, PP4)
2	*DYNC2H1* (NM_001080463.2) c.5682_5683del	p.His1896Tyrfs*9	P	SCV000788364	33.0	—	—	—	—	0.00046	Pathogenic Previously reported (PVS1, PM2, PM3, PP5)
3	*IFT140* (NM_014714.4) c.3602 G > A	p.Arg1201His	M	318004	22.8	0.028 (Benign)	0.152 (Tolerated)	−0.914 (Neutral)	0.010 (Likely Benign)	0.00619	Uncertain Significant (PM2, BP4)
4	*IFT122* (NM_018262) c.457 A > C	p.Asn153His	P	—	25.9	0.771 (Possibly damaging)	—	−3.922 (Deleterious)	0.059 (Possibly Pathogenic)	0.00230	Uncertain Significant (PM2, PP3)

The reference sequences used were *DYNC2H1* (NM_001080463.2), *IFT122* (NM_018262.4), and *IFT140* (NM_014714.4).

*M* maternal, *P* paternal, *CADD* combined annotation-dependent depletion (https://cadd.bihealth.org/), *PolyPhen-2* polymorphism phenotyping v2 (http://genetics.bwh.harvard.edu/pph2), *SIFT* sorting intolerance from tolerant (http://sift-dna.org), *PROVEAN* protein variation effect analyzer (https://www.jcvi.org/research/provean), *M-CAP* Mendelian clinically applicable pathogenicity (http://bejerano.stanford.edu/mcap), # according to gnomAD v2.1.1, *ACMG* American College of Medical Genetics and Genomics, *AMP* Association for Molecular Pathology.

The paternally inherited variant c.5682_5683del of *DYNC2H1* has previously been reported as a pathogenic variant of SRTD type 3 in a compound heterozygous state with c.9070 C > T (p.Arg3004Cys)^15^. In contrast, the maternal variant c.3404 A > T (p.Glu1135Val) has not been reported in affected individuals and is absent in gnomAD (https://gnomad.broadinstitute.org), JMorp (https://jmorp.megabank.tohoku.ac.jp) and our local database (Swedish in-house database, which contains 14,200 cases). Using in silico prediction tools, this missense variant is predicted to be deleterious (Table 1). According to the American College of Medical Genetics and Genomics and the Association for Molecular Pathology (ACMG/AMP) guidelines, this variant is classified as likely pathogenic (PM2, PM3, PP3, and PP4). The other two variants, c.3602 G > A in *IFT140* and c.457 A > C in *IFT122*, are classified as being of uncertain significance (Table 1).

The *DYNC2H1* gene on chromosome 11q22.3 encodes a subunit of the cytoplasmic dynein complex^16^. Homozygous or compound heterozygous *DYNC2H1* pathogenic variants are known to cause SRTD type 3 or short-rib thoracic dysplasia (formerly asphyxiating thoracic dysplasia-Jeune syndrome)^1,3,5,17^. The radiographic presentation of the currently reported fetus was different from that of SRTD3 or short-rib thoracic dysplasia, mainly because of the defect mineralization of the long tubular bones. Triallelic inheritance is a well-recognized phenomenon in ciliopathies^18,19^, and the *IFT122* and *IFT140* variants may have contributed to the severity of the phenotype in this fetus. However, the available evidence is currently insufficient to determine the role of this variant in this disease.

In conclusion, we report an atypical phenotype in a fetus with SRTD with deficient ossification of tubular bones caused by pathogenic variants in the *DYNC2H1* gene.

## HGV database

The relevant data from this Data Report are hosted at the Human Genome Variation Database at 10.6084/m9.figshare.hgv.3471.
